# Increased Expression of Long Noncoding RNA LOC100506314 in T cells from Patients with Nonsegmental Vitiligo and Its Contribution to Vitiligo Pathogenesis

**DOI:** 10.1155/2023/2440377

**Published:** 2023-09-12

**Authors:** Ning-Sheng Lai, Hui-Chun Yu, Hsien-Bin Huang, Hsien-Yu Huang Tseng, Ming-Chi Lu

**Affiliations:** ^1^Division of Allergy, Immunology and Rheumatology, Dalin Tzu Chi Hospital, Buddhist Tzu Chi Medical Foundation, Dalin 62247, Chiayi, Taiwan; ^2^School of Medicine, Tzu Chi University, Hualien City 97071, Taiwan; ^3^Department of Biomedical Sciences, National Chung Cheng University, Minxiong, Chiayi 62130, Taiwan

## Abstract

This study aimed to identify the abnormal expression of long noncoding RNAs (lncRNAs) in T cells from patients with vitiligo and to investigate their functional roles in the immune system. Using microarray analysis, the expression levels of RNA transcripts in T cells from patients with vitiligo and controls were compared. We identified several genes and validated their expression levels in T cells from 41 vitiligo patients and 41 controls. The biological functions of the lncRNAs were studied in a transfection study using an RNA pull-down assay, followed by proteomic analysis and western blotting. The expression levels of 134 genes were significantly increased, and those of 142 genes were significantly decreased in T cells from vitiligo patients. After validation, six genes had increased expression, and three genes had decreased expression in T cells from patients with vitiligo. T-cell expression of LOC100506314 was increased in vitiligo, especially CD4+, but not CD8+ T cells. The expression levels of LOC100506314 in CD4+ T cells was positively and significantly associated with the severity of vitiligo. LOC100506314 was bound to the signal transducer and activator of transcription 3 (STAT3) and macrophage migration inhibitory factor (MIF). Enhanced expression of LOC100506314 inhibited the phosphorylation of STAT3, protein kinase B (AKT), and extracellular signal-regulated protein kinases (ERK), as well as the levels of nuclear protein of p65 and the expression of *IL-6* and *IL-17* in Jurkat cells and T cells from patients with vitiligo. In conclusion, this study showed that the expression of LOC100506314 was elevated in CD4+ T cells from patients with vitiligo and associated the severity of vitiligo. LOC100506314 interacted with STAT3 and MIF and inhibited *IL-6* and *IL-17* expression by suppressing the STAT3, nuclear factor kappa-light-chain-enhancer of activated B cells (NF-*κ*B), AKT, and ERK pathways. Enhanced expression of LOC100506314 in T cells may be a potential treatment strategy for vitiligo.

## 1. Introduction

Vitiligo is a chronic skin depigmenting disease caused by the selective destruction of melanocytes, where autoimmunity plays a critical role [1]. T cells play an important role in the regulation of the immune system; therefore, it is not surprising that dysregulated T cells are involved in the immunopathogenesis of vitiligo [1–3]. Targeting the Janus kinase (JAK)/signal transducer and activator of transcription (STAT) signaling pathway with JAK inhibitors could lead to significant repigmentation in patients with vitiligo [4, 5]. This finding strongly suggests that autoimmunity, especially dysregulated T cells, plays an essential role in the immunopathogenesis of vitiligo.

Long noncoding RNAs (lncRNAs) are noncoding RNA molecules that are more than 200 nucleotides in length. LncRNAs are key regulators of the gene transcription during inflammatory responses [6]. LncRNAs play important physiological roles in the T cells in response to antigenic stimulation, differentiation into effector cells, and cytokine secretion. Their dysregulated expression can contribute to the immunopathogenesis of autoimmunity, chronic inflammation, cancer, and viremia [7]. Our previous studies demonstrated that aberrant expression of the lncRNAs exists in T cells of patients with rheumatoid arthritis and ankylosing spondylitis, which could contribute to the inflammatory responses [8, 9]. Recently, several studies have investigated the aberrant expression of lncRNAs in the pathogenesis of vitiligo using samples from skin biopsies, serum, or peripheral blood mononuclear cells (PBMCs). These aberrantly expressed lncRNAs can participate in the pathogenesis of vitiligo by affecting interleukin- (IL-) 17 production, oxidative stress-mediated melanocyte injury, and the regulation of melanogenesis-related genes [10–16]. However, no studies have focused on the potential aberrantly expressed lncRNAs in T cells from patients with vitiligo.

We hypothesized that aberrantly expressed lncRNAs exist in T cells from patients with vitiligo, and that these lncRNAs could affect the pathways for downstream target molecules to participate in the pathogenesis of vitiligo.

## 2. Materials and Methods

### 2.1. Patients and Controls

Patients aged 20 years and older with a clinician-confirmed diagnosis of nonsegmental vitiligo were recruited from the outpatient department of Dalin Tzu Chi Hospital. Patients diagnosed with the other systemic autoimmune diseases were excluded from this study. Healthy volunteers were recruited as the controls. The study protocol was approved by the institutional review board of the Dalin Tzu Chi Hospital, Buddhist Tzu Chi Medical Foundation (No. B10901014). The study was performed in accordance with the Declaration of Helsinki, and all participants provided informed consent. The sample size for the multiple regression analysis was calculated using G^*∗*^ Power 3.1.9.4 software (Heinrich Heine Düsseldorf University, Düsseldorf, Germany) set to *α* = 0.05, power = 0.90, two tailed, medium effect size (f2) = 0.15, and three predictors. Therefore, the total sample size was estimated at 73 individuals. We increased the sample size by 10% for 82 patients to improve the precision. Therefore, 41 patients with vitiligo and 41 healthy controls were enrolled in the validation phase. The severity of the vitiligo was evaluated by the vitiligo extent score (VES) [17] using an online calculator (https://www.vitiligo-calculator.com/). Patients with active vitiligo were defined as any increment in lesions size or number within the recent 6 months, and the rest patients were classified as stable vitiligo [18].

### 2.2. Purification of T cells

The method for the purification of T cells has been described previously [19]. In brief, heparinized venous blood (20 mL) was mixed with a 2% dextran solution (MW 464,000 Da; Sigma–Aldrich, St. Louis, USA) after sampling. Leukocyte-enriched supernatants were separated using a Ficoll–Hypaque density gradient solution (specific gravity 1.077; Pharmacia Biotech, Uppsala, Sweden). The mononuclear cells were aspirated from the interface after centrifugation. T cells were separated by anti-human CD3-coated magnetic beads and the IMag cell separation system (BD Bioscience, Franklin Lakes, NJ, USA). The purity of T cells is more than 95.6%. Then anti-human CD4-coated magnetic beads and anti-human CD8-coated magnetic beads were used to separate the T cells, CD4+ T cells, or CD8+ T cells by IMag cell separation system (BD Bioscience, Franklin Lakes, NJ, USA).

### 2.3. Microarray Analysis

The gene expression profiles of T cells obtained from three female vitiligo patients aged 44, 46, and 48 years and three female controls aged 45, 47, and 48 years were investigated using the microarray analysis (Welgene Biotech, Taipei, Taiwan) as previously described [20]. We extracted total RNA by the TRIzol reagent (Invitrogen, Carlsbad, CA, USA). The obtained RNA was quantified using an ND-1000 spectrophotometer (Thermo Scientific, Wilmington, DE, USA) at OD260 nm and then quantified using a Bioanalyzer 2100 (Agilent Technology, USA) using an RNA 6000 Labchip kit (Agilent Technologies, Santa Clara, CA, USA). Cyanine 3 (Cy3; Agilent Technologies) dye was used to label the total RNA, and the labeled RNA was hybridized to an Agilent SurePrint G3 Human V2 GE 8 × 60 K Microarray (Agilent Technologies). The results were scanned with an Agilent microarray scanner, and the scanned images were analyzed using Feature extraction 10.5.1.1 software (Agilent Technologies, USA). Quantile normalization was used to normalize the raw signal data and analyze the differentially expressed genes.

### 2.4. Measurement of mRNA Expression Levels by qPCR (Quantitative Polymerase Chain Reaction)

Total RNA from patients with vitiligo and controls was extracted using the Quick-RNA MiniPrep kit (Zymo Research, Irvine, CA, USA) according to the manufacturer's instructions and quantified using a NanoDrop 1000 spectrophotometer (Thermo Fisher Scientific, Waltham, Massachusetts, USA). A one-step RT–PCR kit (TaKaRa, Shiga, Japan) with an ABI Prism 7500 Fast Real-Time PCR system (Applied Biosystems, Waltham, MA, USA) was used to measure mRNA expression levels. The following conditions were used for the quantitative PCR: 42°C for 5 min and 95°C for 10 s for RT, followed by 40 cycles of 95°C for 5 s and 60°C for 34 s. The sequences of the primers were shown in *Supplementary 1*. Relative mRNA expression levels were calculated using the following equation: 39−threshold cycle (Ct) adjusted by the expression of 18S ribosomal RNA. The results of melt curve analysis for the RNA transcript by qPCR was shown in *Supplementary 2*.

### 2.5. Transfection Study

Jurkat cells (American Type Culture Collection, Manassas, Virginia, USA) or T cells from patients with vitiligo were transfected with the plasmid pcDNA3.1, encoding LOC100506314, or empty plasmid by electroporation, as described in our previous study with some modifications [9]. The transfected cells were maintained in RPMI-1640 medium with 10% fetal calf serum at 37°C with 5% CO_2_ for 24 or 48 hr. Cells were harvested by centrifugation and then analyzed for mRNA or protein expression.

### 2.6. Preparation of cell Lysates and Nuclear Extract

A Nuclear Extract Kit (Active Motif, Carlsbad, CA, USA) was used to prepare the nuclear extract after cells lysis by 1% NP-40 (Sigma–Aldrich, St. Louis, MO, USA), with a phosphatase inhibitor cocktail (Thermo Fisher Scientific, Waltham, USA), and a proteinase inhibitor cocktail (Sigma–Aldrich). Bradford assay was used to measure the protein concentrations of the samples.

### 2.7. LOC100506314 Pull-Down Assay

The pull-down assay was performed as previously described [9]. In brief, biotin-labeled LOC100506314 was prepared in vitro by pcDNA3.1-LOC100506314 using T7 RNA polymerase (Ambion Inc., Austin, TX, USA) in the presence of RNA biotin labeling kit (Biotin RNA Labeling Mix Roche, Basel, Switzerland). Biotin-labeled RNAs were transcribed from the empty pcDNA3.1 vector and used as a control. The resulting product was purified using an RNeasy Mini Kit (Qiagen, Hilden, Germany). For pull-down of nuclear proteins, biotinylated LOC100506314 (1 *μ*g) and control (1 *μ*g) were mixed with 1.5 mg of nuclear extract at 4°C for 2 hr, followed by the addition of the washed streptavidin-coupled Dynabeads (60 *μ*L) (Thermo Fisher Scientific). After washing four times with IP buffer, the precipitates were obtained by centrifugation (760 × *g*) at 4°C for 2 min. The precipitated products were subjected to shotgun proteomics LC-MS/MS analysis with a 2D linear ion trap mass spectrometer (Orbitrap Elite ETD; Thermo Fisher Scientific) operated using Xcalibur 2.2 software (Thermo Fisher Scientific). The LC-MS/MS analysis was performed by JetFa Biotech. Co., Ltd. (Taichung, Taiwan).

### 2.8. Western Blot Analysis

Western blot was performed as previously described [21]. The antibodies used for western blot analysis were purchased from Cell Signaling Technology (Danvers, MA, USA), including rabbit monoclonal antibodies against STAT3 (#9133), phospho-STAT3 (#9145), protein kinase R (PKR) (#12297), macrophage migration inhibitory factor (MIF) (#75038), p65 (#8242), protein kinase B (AKT) (#9272), phospho-AKT (#9271), extracellular signal-regulated protein kinases (ERK) (#4695), phospho-ERK (#4370), Taiclone Biotechnology (Taipei, Taiwan), rabbit monoclonal antibodies against *β*-actin (tcba13655), and rabbit polyclonal antibodies against lamin A/C (tcba178).

### 2.9. Statistical Analysis

The Mann–Whitney *U* test or Student's *t*-test were used, as appropriate, to compare different gene expression data from patients with vitiligo and controls. A *p* value < 0.05 was considered statistically significant. Multiple linear regression analysis was used to calculate the regression coefficients and significance among different parameters, adjusting for age and sex, using the Stata software (StataCorp, College Station, TX, USA).

## 3. Results

### 3.1. Differential Expression of the Genes in T cells from Patients with Vitiligo and Controls by Microarray Analysis

**Figure 1:**
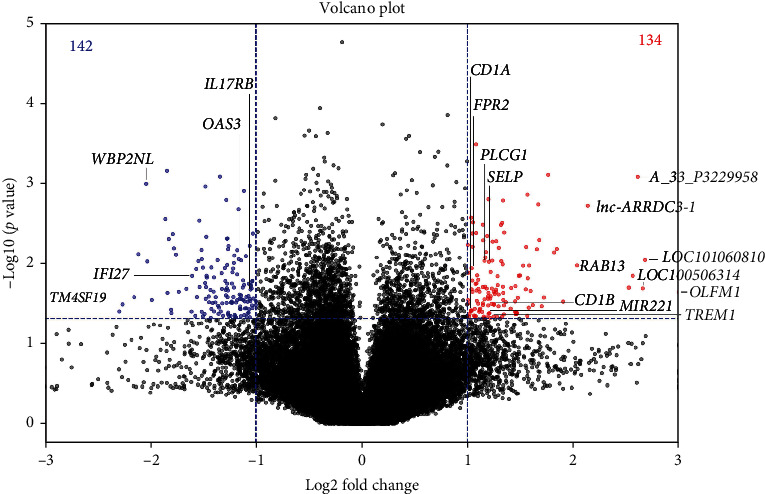
Expression profiles of mRNA transcripts in T cells from three patients with vitiligo and three healthy controls using microarray analysis. Each data point represents the average log10-transformed raw signal intensities of the Cy3-labeled media for each probe in T cells from three patients with vitiligo and three healthy controls.

The gene expression profiles of T cells from three patients with vitiligo and three controls obtained using the microarray analysis are shown in Figure 1. Among these genes, the expression levels of 134 genes were significantly higher, whereas the expression levels of 142 genes were significantly lower in T cells from patients with vitiligo than in controls. For protein-coding genes, the expression levels and potential immunologic functions after literature research, while for lncRNAs, only the expression levels were considered. Finally, 13 genes with increased expression were chosen, including *Lnc-ARRDC3-1*, *PLCG1*, *A_33_P3229958*, *CD1A*, *FPR2*, *CD1B*, *OLFM1*, *SELP*, *MIR221*, *TERM1*, *RAB13*, *LOC100506314*, and *LOC101060810*, and five genes with decreased expression in T cells from patients with vitiligo, including *TM4SF19*, *WBP2NL*, *IFI27*, *IL17RB*, and *OAS3*.

### 3.2. Validation of Aberrant Gene Expression in T cells from Patients with Vitiligo and Healthy Controls

A total of 41 patients with vitiligo were recruited from the ambulatory medical services department, and 41 healthy volunteers served as the control group. The demographic data of the patients with vitiligo and the controls are shown in Table 1. Neither age nor sex differed significantly between groups.

**Figure 2:**
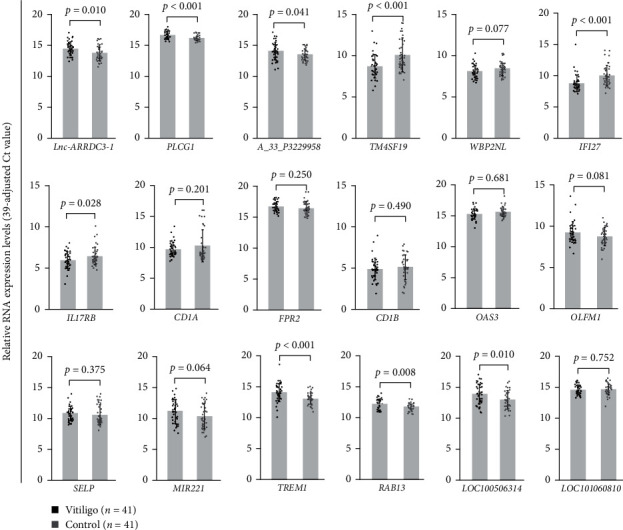
Validation of aberrantly expressed mRNA transcripts in T cells from another 41 patients with vitiligo and 41 healthy controls using quantitative polymerase chain reaction. After adjusted for age and sex, T cells from patients with vitiligo remained significantly elevated in the expression levels of *Lnc-ARRDC3-1* (1.61-fold; *p*=0.011), *PLCG1* (1.37-fold; *p* < 0.001), *A_33_P3229958* (1.46-fold; *p*=0.045), *TERM1* (2.04-fold; *p* < 0.001), *RAB13* (1.36-fold; *p*=0.009), and *LOC100506314* (1.85-fold; *p*=0.011), and decreased in the expression levels of *TM4SF19* (0.38-fold; *p* < 0.001), *IFI27* (0.43-fold; *p*=0.001), and *IL17RB* (0.70-fold; *p*=0.027) compared to those in controls. *P* values were obtained by *t*-test.

As shown in Figure 2, we found that six genes, *Lnc-ARRDC3-1* (1.61-fold; *p*=0.010), *PLCG1* (1.37-fold; *p* < 0.001), *A_33_P3229958* (1.47-fold; *p*=0.041), *TERM1* (2.03-fold; *p* < 0.001), *RAB13* (1.36-fold; *p*=0.008), and *LOC100506314* (1.86-fold; *p*=0.010) were upregulated, and three genes, *TM4SF19* (0.38-fold; *p* < 0.001), *IFI27* (0.42-fold; *p* < 0.001), and *IL17RB* (0.70-fold; *p*=0.028) were downregulated in T cells from patients with vitiligo. After adjusting for age and sex, T cells from patients with vitiligo remained significantly elevated in the expression levels of *Lnc-ARRDC3-1* (1.61-fold; *p*=0.011), *PLCG1* (1.37-fold; *p* < 0.001), *A_33_P3229958* (1.46-fold; *p*=0.045), *TERM1* (2.04-fold; *p* < 0.001), *RAB13* (1.36-fold; *p*=0.009), and *LOC100506314* (1.85-fold; *p*=0.011), and decreased in the expression levels of *TM4SF19* (0.38-fold; *p* < 0.001), *IFI27* (0.43-fold; *p*=0.001), and *IL17RB* (0.70-fold; *p*=0.027) compared to those in controls.

### 3.3. Correlations between the Expression Levels of the mRNA Transcripts with Demographic Data of Patients with Vitiligo

As shown in Table 2, there was no statistically significant correlation among the demographic data of patients with vitiligo, including age, sex, and duration of diagnosis, and the expression levels of *Lnc-ARRDC3-1*, *PLCG1*, *A_33_P3229958*, *TERM1*, *RAB13*, *LOC100506314*, *TM4SF19*, *IFI27*, and *IL17RB* in patients with vitiligo (*n* = 41). Among the lncRNAs, the differential expression levels were the greatest for *LOC100506314*, therefore, we chose *LOC100506314* for further study.

### 3.4. Expression Levels of LOC100506314 in CD4+ or CD8+ T cells and Its Correlation with Disease Activity and Severity

**Figure 3:**
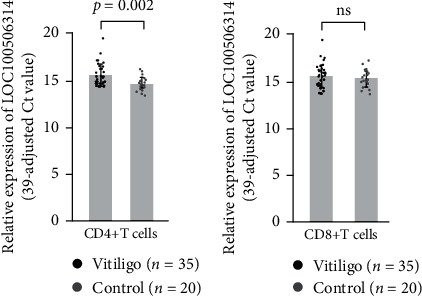
Expression levels of LOC100506314 in CD4+ and CD8+ T cells from patients with vitiligo compared to those from the controls. (a) CD4+ T cells and (b) CD8+ T cells.

We found that the expression of *LOC100506314* in significantly elevated in CD4+ T cells (1.94-fold; *p*=0.002; Figure 3(a)), but not CD8+ T cells (1.18-fold, *p*=0.576; Figure 3(b)) from patients with vitiligo compared with the controls. After adjusted for age and sex, the expression of *LOC100506314* in still significantly elevated in CD4+ T cells (*p*=0.019).

In Table 3, we found that the expression levels of *LOC100506314* in CD4+ T cells were significantly associated with the severity, but not the activity of vitiligo in the univariate analysis. After adjusting for sex, age, and duration of disease, the severity of vitiligo measured by VES was positively, statistically significantly associated with the expression levels of *LOC100506314* in CD4+ T cells (*p*=0.012).

### 3.5. Search and Validation of the Proteins Interact with LOC100506314

**Figure 4:**
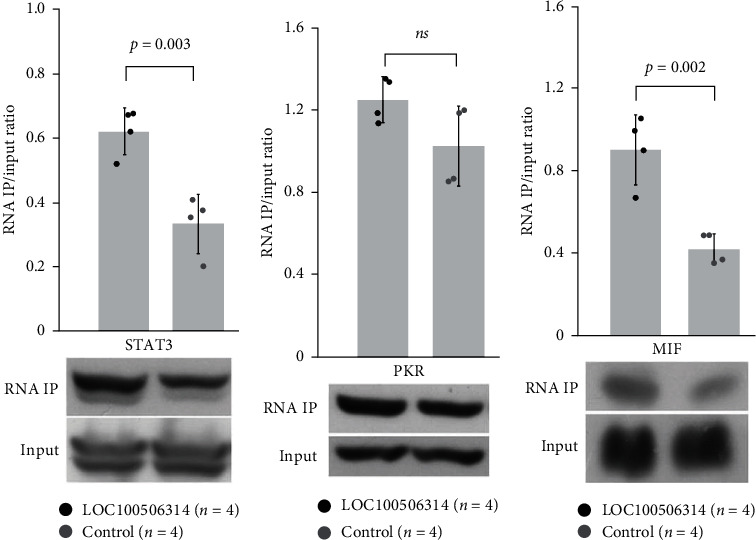
Validation of the proteins that interacted with LOC100506314. RNA pull-down assay was performed with the biotinylated LOC100506314. The biotin-labeled LOC100506314 was prepared in vitro by pcDNA3.1LOC100506314 using T7 RNA polymerase in the presence of the kit for RNA biotin labeling. An empty vector of pcDNA3.1 was used as a control. The extracted nuclear proteins were incubated with the biotin-conjugated LOC100506314 or control, followed by a pull-down with streptavidin-conjugated beads. The precipitated proteins were subjected to analysis by shotgun proteomics analysis. We chose three proteins, including (a) Signal transducer and activator of transcription 3 (STAT3), (b) Protein kinase R (PKR), and (c) macrophage migration inhibitory factor (MIF) that related to the immune response for further validation by western blotting.

LncRNAs often exert their functions by binding to one or more proteins. The interaction of protein–lncRNAs is an important mechanism in the regulation of cell functions, and their dysregulation can also contribute to the pathology of diseases [22]. We searched for potential binding proteins of LOC100506314. After RNA immunoprecipitation and proteomic analysis, we found that 87 proteins were precipitated only by biotinylated LOC100506314 (*Supplementary 1*). After reviewing the literature, we selected three proteins, STAT3, PKR, and MIF, which are related to the immune response, for further validation by western blotting. We confirmed that LOC100506314 interacts with STAT3 and MIF, but not with PKR (Figure 4). All the experiments were repeated four times.

### 3.6. Effect of LOC100506314 Overexpression in Jurkat Cells on Downstream Signaling Pathway of STAT3 and MIF

**Figure 5:**
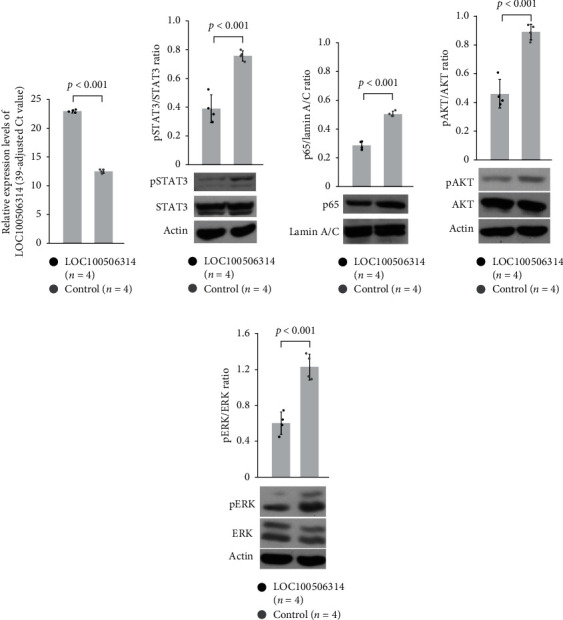
Effect of LOC100506314 overexpression in jurkat cells on downstream signaling pathway of STAT3 and MIF. LOC100506314 was overexpressed in Jurkat cells using transfection with plasmid encoding LOC100506314 and an empty plasmid was used for the control. (a) The transfection of plasmid encoding LOC100506314 effectively increased the expression of LOC100506314 in jurkat cells. (b) The transfection of LOC100506314 suppressed the phosphorylation of STAT3. (c) The transfection of LOC100506314 decreased the nuclear protein levels of p65. (d) The transfection of LOC100506314 decreased the phosphorylation of AKT. (e) The phosphorylation of ERK was also suppressed after the transfection of LOC100506314.

Since LOC100506314 could bind to STAT3 and MIF, we speculated that the increased expression of LOC100506314 could impair the downstream signaling pathways of STAT3 and MIF. We validated that LOC100506314 was overexpressed in Jurkat cells after transfection with a plasmid encoding LOC100506314 (Figure 5(a)). An empty plasmid was used as a control. We found that the STAT3 phosphorylation ratio decreased in LOC100506314 overexpressed Jurkat cells compared to controls (Figure 5(b)). It has been reported that pSTAT3 can interact with p65, one of the components of NF-*κ*B (nuclear factor kappa-light-chain-enhancer of activated B cells) [23]. In Figure 5(c), we found that the protein levels of p65 in the nuclear extract also decreased in LOC100506314 overexpressed Jurkat cells. MIF has been reported to activate cells via ERK and AKT [24]. We confirmed that the phosphorylation ratio of ERK and AKT decreased in LOC100506314 overexpressed Jurkat cells compared with that in the controls (Figures 5(d) and 5(e)). All the experiments were repeated four times.

### 3.7. Effect of LOC100506314 Overexpression in Jurkat Cells on Cytokine Expression

**Figure 6:**
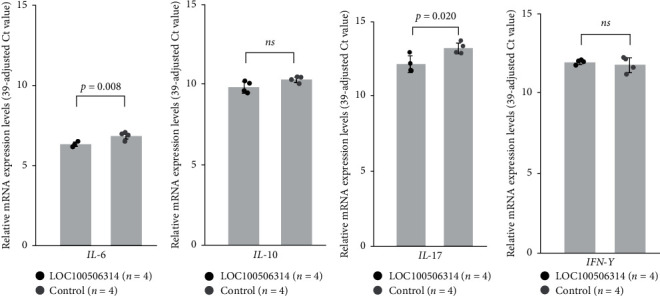
Effect of LOC100506314 overexpression in Jurkat cells on the expression of cytokines, including (a) *IL-6*, (b) *IL-10*, (c) *IL-17*, and (d) *IFN-γ*.

We found that the expression levels of *IL-6* (0.7-fold) and *IL-17* (0.48-fold), but not *IL-10* or *interferon-γ* (*IFN-γ*), were decreased in LOC100506314-overexpressed Jurkat cells compared with those in the controls (Figure 6). All the experiments were repeated four times.

### 3.8. Effect of LOC100506314 Overexpression in T cells from Patients with Vitiligo on Cytokine Expression

**Figure 7:**
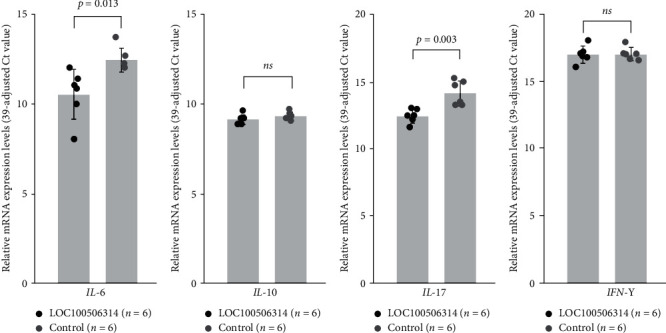
Effect of LOC100506314 overexpression in T cells from patients with vitiligo on the expression of cytokines, including (a) *IL-6*, (B)b *IL-10*, (c) *IL-17*, and (d) *IFN-γ*.

Finally, we found that expression levels of *IL-6* (0.27-fold) and *IL-17* (0.30-fold), but not *IL-10* or *IFN-γ*, were also decreased in T cells from patients with vitiligo (*n* = 6) compared with the controls (*n* = 6), after the transfection of plasmid encoding LOC100506314 (Figure 7).

### 3.9. A Summary Graphic for the Roles of Abnormal T-Cell Expression of LOC100506314 in the Immunopathogenesis of Vitiligo

**Figure 8:**
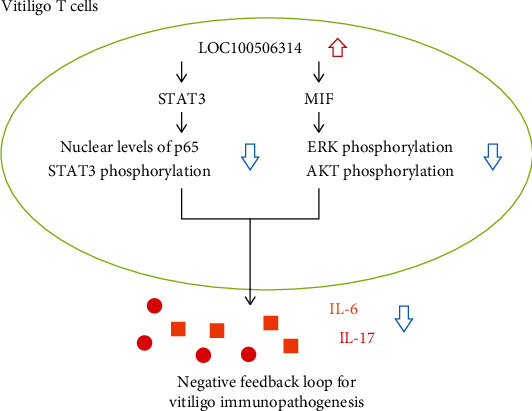
Roles of abnormal T-cell expression of LOC100506314 in the immune pathogenesis of vitiligo. The expression levels of LOC100506314 increased in T cells from patients with vitiligo. LOC100506314 could bind STAT3 and MIF and suppressed STAT3, AKT, and ERK phosphorylation and decreased nuclear protein levels of p65, resulting decreased expression of IL-6 and IL-17. Therefore, LOC100506314 appeared to play a negative feedback role for vitiligo immune pathogenesis.

A representative graph showed how the increased expression of LOC100506314 in T cells participated to the immunopathogenesis of vitiligo (Figure 8).

## 4. Discussion

In this study, we found that among lncRNAs, the expression of LOC100506314 was increased in T cells from patients with vitiligo and participated in the immunopathogenesis of vitiligo via binding to STAT3 and MIF, which could affect its downstream signaling. We noted that other genes, such as *Lnc-ARRDC3-1*, *PLCG1*, *A_33_P3229958*, *TERM1*, and *RAB13*, showed increased expression, whereas *TM4SF19*, *IFI27*, and *IL17RB* showed decreased expression in T cells from patients with vitiligo compared with the controls. Among these genes, *IFI27* was upregulated when keratinocytes were stimulated with IFN-*γ*, one of the critical cytokines for disease progression and autoreactive T-cell homing to the epidermis [25]. The roles of other genes in the pathogenesis of vitiligo and their function in T cells need further investigation.

We found that LOC100506314 could bind to STAT3 and MIF and affect their downstream signaling with the inhibition STAT3 phosphorylation and nuclear levels of p65 for STAT3 signaling pathway and ERK and AKT phosphorylation for MIF signaling pathway. Samaka et al. [26] found that STAT3 was overexpressed in vitiligo skin lesions and that the dermal expression of STAT3 was positively correlated with the dermal expression of JAK1. Increased STAT3 activation can lead to skin Th17 cell infiltration [27]. Targeting STAT3 with miR-21-5p can decrease apoptosis in melanocytes and increase tyrosinase activity [28], which could be a potential treatment for vitiligo. Most importantly, small-molecule JAK inhibitors that target the JAK/STAT pathway are emerging treatments for vitiligo [29]. However, it should be noted that there are many known side effects, such as herpes zoster, opportunistic infections, and a few potential side effects, including venous thromboembolism and malignancy, associated with the use of JAK inhibitors [30]. Thus, new therapeutic approaches, such as enhancing the expression of LOC100506314, are required.

MIF is a cytokine that plays an important role in the regulation of innate immunity, including the host's antimicrobial alarm system and stress response that promotes the pro-inflammatory functions of immune cells [31]. Traditionally, MIF acts in an autocrine and paracrine manner by binding and activating receptors; however, MIF has been shown to physically interact with various intracellular proteins and to affect the biological function of cells [32]. MIF could also contribute to vitiligo pathogenesis. Ma et al. [33] demonstrated that serum MIF concentrations and mRNA levels were significantly higher in the PBMCs from patients with vitiligo than in the controls. In addition, the severity of vitiligo was positively correlated with serum MIF concentrations and *MIF* mRNA levels in PBMCs [33]. *MIF* polymorphisms can also increase the risk of developing vitiligo [34]. The downstream signaling pathway of MIF, including AKT and ERK, was also involved in the pathogenesis of vitiligo [35, 36]. Finally, the transfection of LOC100506314 effectively suppressed the expression of *IL-6* and *IL-17* in T cells from patients with vitiligo, which are known to be elevated in the serum of patients with vitiligo [37, 38]. As expected, the expression of LOC100506314 was elevated CD4+ T cells from patients with vitiligo and associated the severity of vitiligo. In addition, regulatory T cells (Tregs) play a crucial role in the pathogenesis of vitiligo, and patients with vitiligo had decreased Tregs frequency and function [39–41]. Inhibition of STAT3 phosphorylation via LOC100506314 might facilitate the differentiation of Tregs [42]. We speculated that LOC100506314 could play a negative feedback role in the inflammatory responses by suppressing MIF and STAT3 expression, leading to decreased STAT3 and NF-*κ*B activation, subsequently decreasing proinflammatory cytokine expression. The increased expression of LOC100506314 in T cells from patients with vitiligo could be due to an insufficient negative feedback pathway of LOC100506314 in patients with vitiligo. The activation of STAT3 and NF-*κ*B not only plays an important role in vitiligo, but also acts as an inflammation amplifier in many immune diseases [43]. Furthermore, STAT3 and NF-*κ*B are required for communication between cancer cells and their microenvironment, mainly with inflammatory/immune cells that infiltrate the tumors. Therefore, strategies to increase LOC100506314 expression may represent a new direction in the treatment of vitiligo. Increasing LOC100506314 expression can also be a potential strategy to control inflammation and inhibit carcinogenesis [44].

There were two limitations to our study. First, the biological effects of LOC100506314 were assessed at the cellular level. Animal studies are needed to clarify the biological effects of LOC100506314 on the immune system. Second, we did not investigate the expression of LOC100506314 in tissue from patients with vitiligo.

## 5. Conclusions

Using microarray analysis, we found that the expression levels of LOC100506314 increased in CD4+ T cells from patients with vitiligo and correlated with the severity of vitiligo. Using a transfection study, we found that LOC100506314 could bind STAT3 and MIF. Enhanced expression of LOC100506314 suppressed STAT3, AKT, and ERK phosphorylation and nuclear protein levels of p65, which are known to participate in the pathogenesis of vitiligo. Increased expression of LOC100506314 in T cells could be a potential therapeutic strategy for the treatment of vitiligo.

## Figures and Tables

**Table 1:** Demographics of patients with vitiligo and healthy volunteers.

Variable	Healthy volunteers (*N* = 41)	Patients with vitiligo (*N* = 41)	*p* Value
Age (mean years ± SD)	37.0 ± 5.1	37.4 ± 9.0	0.807
Sex (female : male)	23 : 18	23 : 18	>0.999
Duration of disease (mean years ± SD)		6.4 ± 6.0	
Classification			
Nonsegmental vitiligo		100% (41/41)	
Segmental vitiligo		0% (0/41)	

*Note*: SD, standard deviation.

**Table 2:** Regression analyses assessing the correlations between the expression levels of the mRNA transcripts with demographic data of patients with vitiligo.

Variables	Duration of disease (per year)	Sex (male/female)	Age (per 10 year)
*Lnc-ARRDC3-1*	1.03 (0.99–1.07)	0.72 (0.45–1.16)	1.14 (0.87–1.51)
*PLCG1*	1.01 (0.99–1.04)	0.91 (0.69–1.21)	1.04 (0.89–1.12)
*A_33_P3229958*	1.05 (0.99–1.10)	0.72 (0.41–1.42)	1.23 (0.87–1.75)
*TM4SF19*	1.03 (0.98–1.09)	1.63 (0.87–3.03)	1.12 (0.98–1.05)
*IFI27*	0.97 (0.93–1.03)	0.86 (0.47–1.60)	0.93 (0.66–1.32)
*IL17RB*	1.03 (0.99–1.07)	1.02 (0.65–1.58)	0.93 (0.72–1.19)
*TERM1*	1.02 (0.96–1.08)	0.66 (0.34–1.26)	1.04 (0.71–1.52)
*RAB13*	1.00 (0.97–1.04)	1.05 (0.71–1.56)	1.07 (0.86–1.34)
*LOC100506314*	1.05 (0.99–1.11)	0.68 (0.33–1.37)	1.37 (0.92–2.03)

*Note*: Data are presented as fold change with 95% confidence interval. All *p* values were >0.05.

**Table 3:** Univariate and multivariate regression analyses assessing the correlations between the severity and activity of vitiligo with *LOC100506314* expression levels in CD4+ T cells.

	Univariate		Multivariate	
Variables	Fold change	*p* Value	Fold change	*p* Value
Age (per 10 year)	0.94 (0.72–1.23)	0.652	0.97 (0.75–1.22)	0.737
Sex (male/female)	0.64 (0.37–1.10)	0.104	0.77 (0.45–1.34)	0.341
Duration of disease (per year)	0.98 (0.95–1.00)	0.075	0.98 (0.95–1.00)	0.092
VES	**1.08 (1.01–1.15)**	**0.029**	**1.09 (1.02–1.16)**	**0.012**
Activity (active/stable)	0.77 (0.36–1.63)	0.478	–	–

*Note*: Data are presented as fold change with 95% confidence interval. VES, vitiligo extent score. Bold value represents *p* < 0.05.

## Data Availability

Microarray data had been uploaded in gene expression omnibus (GEO) of the National Center for Biotechnology Information and can be accessed (GEO: GSE205751). The original contributions presented in the study are included in the supplementary material. The datasets analyzed during the current study are available from the corresponding authors on reasonable request.

## Supplementary Materials

Supplementary 1 Table S1: the LOC100506314 pull-down proteins. Table S2: the LOC100506314 pull-down proteins.

Supplementary 2 Melt curve analysis for Lnc-ARRDC3-1, PLCG1, A_33_P3229958, TM4SF19, WBP2NL, IFI27, IL17RB, CD1A, FPR2, CD1B, OAS3, OLFM1, SELP, MIR221, TERM1, RAB13, LOC100506314, and LOC101060810 transcripts. Representative image for melt curve analysis for (A) Lnc-ARRDC3-1 transcripts, (B) PLCG1 transcripts, (C) A_33_P3229958 transcripts, (D) TM4SF19 transcripts, (E) WBP2NL transcripts, (F) IFI27 transcripts, (G) IL17RB transcripts, (H) CD1A transcripts, (I) FPR2 transcripts, (J) CD1B transcripts, (K) OAS3 transcripts, (L) OLFM1 transcripts, (M) SELP transcripts, (N) MIR221 transcripts, (O) TERM1 transcripts and (P) RAB13 transcripts, (Q) LOC100506314 transcripts, and (R) LOC101060810 transcripts.
