# Supplementary material for: Increased Expression of Long Noncoding RNA LOC100506314 in T cells from Patients with Nonsegmental Vitiligo and Its Contribution to Vitiligo Pathogenesis
Source: Mediators Inflamm. 2023 Sep 12;2023:2440377. doi: 10.1155/2023/2440377 (PMC10509001; doi:10.1155/2023/2440377)
Supplement: Supplementary 1 — Table S1: the LOC100506314 pull-down proteins. Table S2: the LOC100506314 pull-down proteins. [file 2440377.f1.doc]

**Table S1:** **Primer sequences for qPCR analysis**

| **Primer sequence for qPCR**  **analysis of**  **Lnc-ARRDC3-1, PLCG1, A_33_P3229958, CD1A, FPR2, CD1B, OLFM1, SELP, MIR221, TERM1, RAB13, LOC100506314, LOC101060810, TM4SF19, WBP2NL, IFI27, IL17RB, and OAS3.** | | |
| --- | --- | --- |
| **Gene** | **Primer** | **Primer sequence** |
| ***Lnc-ARRDC3-1*** | Forward Primer | 5’-GAA GGA AGA AAG GAG GGC GG-3’ |
|  | Reverse Primer | 5’-GTG ATC AGC CCG CTT AGG C-3’ |
| ***PLCG1*** | Forward Primer | 5’-CTG TCC CAG GTC AAC TAC CG-3’ |
|  | Reverse Primer | 5’-TAC ATG AGG CTG CGG TAC AGC-3’ |
| ***A_33_P3229958*** | Forward Primer | 5’-GCT ACC AGA CAT GCA GCC TG-3’ |
|  | Reverse Primer | 5’-ACA GAG TAC TAT TAC AGT ACC G-3’ |
| ***CD1A*** | Forward Primer | 5’-AAC CCT GGA GGT GGC CGC-3’ |
|  | Reverse Primer | 5’-AAG GCA CTA TCA CCG CCA AG-3’ |
| ***FPR2,*** | Forward Primer | 5’-TCC TGC ATC CAG TCT GGG C-3’ |
|  | Reverse Primer | 5’-GAA AGT AGA GTA TGT GTC CCC-3’ |
| ***CD1B*** | Forward Primer | 5’-AAG AAG CCC AAG ATA TCA GCC-3’ |
|  | Reverse Primer | 5’-ATC ACA CTG TTA GTA CAG TCT T-3’ |
| ***OLFM1*** | Forward Primer | 5’-CAG TGT TCA CCC TTA TAG AGA C-3’ |
|  | Reverse Primer | 5’-GGC GAG CCC TTC CTA TCT GAG-3’ |
| ***SELP*** | Forward Primer | 5’-TGA ATA CGT GAG AGA GTG TGG-3’ |
|  | Reverse Primer | 5’-CCA GAA GCC AAG CAT TCC AG-3’ |
| ***MIR221*** | Forward Primer | 5’-TGA ACA TCC AGG TCT GGG AC-3’ |
|  | Reverse Primer | 5’-GAG AAC ATG TTT CCA GGT AGC-3’ |
| ***TERM1*** | Forward Primer | 5’-TCC TAC CAC CAC TAA GGC C-3’ |
|  | Reverse Primer | 5’-CCG GAA CCC TGA TGA TAT CTG-3’ |
| ***RAB13*** | Forward Primer | 5’-CGA GAA TAT TCA GAA CTA GAT G-3’ |
|  | Reverse Primer | 5’-ACT TAT CGG CCT GCT CCT TC-3’ |
| ***LOC100506314*** | Forward Primer | 5’-CAA TGG ATC AGC ACA CAC TGG-3’ |
|  | Reverse Primer | 5’-CAC AGG TCC AGC CTG GTT G-3’ |
| ***LOC101060810*** | Forward Primer | 5’-GGA GAG AAA CCC TAC AAA TGT G-3’ |
|  | Reverse Primer | 5’-AGG TTG AAG ACC AGT TAT AAG C-3’ |
| ***TM4SF19*** | Forward Primer | 5’-GCT GCT GGG GCC AAC GTG-3’ |
|  | Reverse Primer | 5’-CAG CCC ATC AAG GAG ATG AG-3’ |
| ***WBP2NL*** | Forward Primer | 5’-GAG AAC CGC CGC GGA GCC-3’ |
|  | Reverse Primer | 5’-GAT CAC TGA TGG AGC ATG AAG-3’ |
| ***IFI27*** | Forward Primer | 5’-CTC ACC TCA TCA GCA GTG AC-3’ |
|  | Reverse Primer | 5’-ATG GAG GAC GAG GCG ATT CC-3’ |
| ***IL17RB*** | Forward Primer | 5’-CCT GTA GAG CTG AAC ACA GTC-3’ |
|  | Reverse Primer | 5’-ACA GGC TTC CGG CGT TGA C-3’ |
| ***OAS3*** | Forward Primer | 5’-TGA CTG TGT ATG CCT GGG AG-3’ |
|  | Reverse Primer | 5’-CCA ACA GTC TTG TCC TTG GC-3’ |

Table S2: The LOC100506314 pull-down proteins

| Accession | Description | MW [kDa] | Σ# PSMs | Protein Area | | #PSM | | Normalized PSM | |
| --- | --- | --- | --- | --- | --- | --- | --- | --- | --- |
|  |  |  |  | Neg | Pos | Neg | Pos | Neg | Pos |
| Q9BQE3 | Tubulin alpha-1C chain OS=Homo sapiens OX=9606 GN=TUBA1C PE=1 SV=1 - [TBA1C_HUMAN] | 49.9 | 151 | 7.652E8 | 6.443E8 |  | 76 | - | 78.6 |
| P14174 | Macrophage migration inhibitory factor OS=Homo sapiens OX=9606 GN=MIF PE=1 SV=4 - [MIF_HUMAN] | 12.5 | 4 | 1.012E8 | 3.925E7 |  | 4 | - | 4.1 |
| P11021 | Endoplasmic reticulum chaperone BiP OS=Homo sapiens OX=9606 GN=HSPA5 PE=1 SV=2 - [BIP_HUMAN] | 72.3 | 10 | 6.816E7 | 5.195E7 |  | 6 | - | 6.2 |
| P62753 | 40S ribosomal protein S6 OS=Homo sapiens OX=9606 GN=RPS6 PE=1 SV=1 - [RS6_HUMAN] | 28.7 | 6 | 6.073E7 | 2.996E7 |  | 6 | - | 6.2 |
| Q05519 | Serine/arginine-rich splicing factor 11 OS=Homo sapiens OX=9606 GN=SRSF11 PE=1 SV=1 - [SRS11_HUMAN] | 53.5 | 3 | 3.390E7 | 1.051E7 |  | 3 | - | 3.1 |
| O15145 | Actin-related protein 2/3 complex subunit 3 OS=Homo sapiens OX=9606 GN=ARPC3 PE=1 SV=3 - [ARPC3_HUMAN] | 20.5 | 4 | 3.296E7 | 2.258E7 |  | 4 | - | 4.1 |
| P49711 | Transcriptional repressor CTCF OS=Homo sapiens OX=9606 GN=CTCF PE=1 SV=1 - [CTCF_HUMAN] | 82.7 | 3 | 3.295E7 | 2.286E7 |  | 3 | - | 3.1 |
| Q96GM5 | SWI/SNF-related matrix-associated actin-dependent regulator of chromatin subfamily D member 1 OS=Homo sapiens OX=9606 GN=SMARCD1 PE=1 SV=2 - [SMRD1_HUMAN] | 58.2 | 4 | 2.963E7 | 1.140E7 |  | 3 | - | 3.1 |
| Q9NZI8 | Insulin-like growth factor 2 mRNA-binding protein 1 OS=Homo sapiens OX=9606 GN=IGF2BP1 PE=1 SV=2 - [IF2B1_HUMAN] | 63.4 | 4 | 2.351E7 | 2.240E7 |  | 4 | - | 4.1 |
| P30086 | Phosphatidylethanolamine-binding protein 1 OS=Homo sapiens OX=9606 GN=PEBP1 PE=1 SV=3 - [PEBP1_HUMAN] | 21.0 | 3 | 2.346E7 | 8.540E6 |  | 3 | - | 3.1 |
| Q92879 | CUGBP Elav-like family member 1 OS=Homo sapiens OX=9606 GN=CELF1 PE=1 SV=2 - [CELF1_HUMAN] | 52.0 | 4 | 2.184E7 | 2.553E7 |  | 4 | - | 4.1 |
| Q9NV88 | Integrator complex subunit 9 OS=Homo sapiens OX=9606 GN=INTS9 PE=1 SV=2 - [INT9_HUMAN] | 73.8 | 3 | 2.169E7 | 1.012E7 |  | 3 | - | 3.1 |
| Q14739 | Delta(14)-sterol reductase LBR OS=Homo sapiens OX=9606 GN=LBR PE=1 SV=2 - [LBR_HUMAN] | 70.7 | 4 | 2.127E7 | 2.273E7 |  | 4 | - | 4.1 |
| P23193 | Transcription elongation factor A protein 1 OS=Homo sapiens OX=9606 GN=TCEA1 PE=1 SV=2 - [TCEA1_HUMAN] | 33.9 | 2 | 2.092E7 | 1.275E7 |  | 2 | - | 2.1 |
| P09234 | U1 small nuclear ribonucleoprotein C OS=Homo sapiens OX=9606 GN=SNRPC PE=1 SV=1 - [RU1C_HUMAN] | 17.4 | 4 | 2.027E7 | 2.374E7 |  | 4 | - | 4.1 |
| Q13618 | Cullin-3 OS=Homo sapiens OX=9606 GN=CUL3 PE=1 SV=2 - [CUL3_HUMAN] | 88.9 | 3 | 1.779E7 | 1.171E7 |  | 3 | - | 3.1 |
| O95782 | AP-2 complex subunit alpha-1 OS=Homo sapiens OX=9606 GN=AP2A1 PE=1 SV=3 - [AP2A1_HUMAN] | 107.5 | 6 | 1.716E7 | 1.226E7 |  | 4 | - | 4.1 |
| O75083 | WD repeat-containing protein 1 OS=Homo sapiens OX=9606 GN=WDR1 PE=1 SV=4 - [WDR1_HUMAN] | 66.2 | 2 | 1.683E7 | 1.876E7 |  | 2 | - | 2.1 |
| Q13247 | Serine/arginine-rich splicing factor 6 OS=Homo sapiens OX=9606 GN=SRSF6 PE=1 SV=2 - [SRSF6_HUMAN] | 39.6 | 4 | 1.669E7 | 1.247E7 |  | 2 | - | 2.1 |
| O43488 | Aflatoxin B1 aldehyde reductase member 2 OS=Homo sapiens OX=9606 GN=AKR7A2 PE=1 SV=3 - [ARK72_HUMAN] | 39.6 | 3 | 1.657E7 | 1.009E7 |  | 3 | - | 3.1 |
| Q13620 | Cullin-4B OS=Homo sapiens OX=9606 GN=CUL4B PE=1 SV=4 - [CUL4B_HUMAN] | 103.9 | 12 | 1.555E7 | 9.539E6 |  | 6 | - | 6.2 |
| P16401 | Histone H1.5 OS=Homo sapiens OX=9606 GN=H1-5 PE=1 SV=3 - [H15_HUMAN] | 22.6 | 3 | 1.487E7 | 5.414E6 |  | 3 | - | 3.1 |
| P61964 | WD repeat-containing protein 5 OS=Homo sapiens OX=9606 GN=WDR5 PE=1 SV=1 - [WDR5_HUMAN] | 36.6 | 4 | 1.449E7 | 1.710E7 |  | 4 | - | 4.1 |
| Q9NV06 | DDB1- and CUL4-associated factor 13 OS=Homo sapiens OX=9606 GN=DCAF13 PE=1 SV=2 - [DCA13_HUMAN] | 51.4 | 2 | 1.420E7 | 1.775E7 |  | 2 | - | 2.1 |
| Q9NQ29 | Putative RNA-binding protein Luc7-like 1 OS=Homo sapiens OX=9606 GN=LUC7L PE=1 SV=1 - [LUC7L_HUMAN] | 43.7 | 10 | 1.392E7 | 8.744E6 |  | 6 | - | 6.2 |
| Q9UFW8 | CGG triplet repeat-binding protein 1 OS=Homo sapiens OX=9606 GN=CGGBP1 PE=1 SV=2 - [CGBP1_HUMAN] | 18.8 | 2 | 1.284E7 | 9.063E6 |  | 2 | - | 2.1 |
| Q92804 | TATA-binding protein-associated factor 2N OS=Homo sapiens OX=9606 GN=TAF15 PE=1 SV=1 - [RBP56_HUMAN] | 61.8 | 8 | 1.276E7 | 2.153E7 |  | 6 | - | 6.2 |
| Q96CS3 | FAS-associated factor 2 OS=Homo sapiens OX=9606 GN=FAF2 PE=1 SV=2 - [FAF2_HUMAN] | 52.6 | 3 | 1.239E7 | 9.769E6 |  | 3 | - | 3.1 |
| P19525 | Interferon-induced, double-stranded RNA-activated protein kinase OS=Homo sapiens OX=9606 GN=EIF2AK2 PE=1 SV=2 - [E2AK2_HUMAN] | 62.1 | 3 | 1.184E7 | 6.045E6 |  | 3 | - | 3.1 |
| Q8WX92 | Negative elongation factor B OS=Homo sapiens OX=9606 GN=NELFB PE=1 SV=1 - [NELFB_HUMAN] | 65.7 | 3 | 1.171E7 | 9.131E6 |  | 3 | - | 3.1 |
| Q8IX01 | SURP and G-patch domain-containing protein 2 OS=Homo sapiens OX=9606 GN=SUGP2 PE=1 SV=2 - [SUGP2_HUMAN] | 120.1 | 2 | 1.141E7 | 1.194E7 |  | 2 | - | 2.1 |
| Q8TCJ2 | Dolichyl-diphosphooligosaccharide--protein glycosyltransferase subunit STT3B OS=Homo sapiens OX=9606 GN=STT3B PE=1 SV=1 - [STT3B_HUMAN] | 93.6 | 2 | 1.131E7 | 1.623E7 |  | 2 | - | 2.1 |
| P26196 | Probable ATP-dependent RNA helicase DDX6 OS=Homo sapiens OX=9606 GN=DDX6 PE=1 SV=2 - [DDX6_HUMAN] | 54.4 | 3 | 1.098E7 | 1.400E7 |  | 3 | - | 3.1 |
| Q53H12 | Acylglycerol kinase, mitochondrial OS=Homo sapiens OX=9606 GN=AGK PE=1 SV=2 - [AGK_HUMAN] | 47.1 | 3 | 1.006E7 | 2.345E7 |  | 3 | - | 3.1 |
| Q9UIA9 | Exportin-7 OS=Homo sapiens OX=9606 GN=XPO7 PE=1 SV=3 - [XPO7_HUMAN] | 123.8 | 2 | 1.000E7 | 3.004E6 |  | 2 | - | 2.1 |
| P15153 | Ras-related C3 botulinum toxin substrate 2 OS=Homo sapiens OX=9606 GN=RAC2 PE=1 SV=1 - [RAC2_HUMAN] | 21.4 | 8 | 9.950E6 | 1.208E7 |  | 6 | - | 6.2 |
| Q99460 | 26S proteasome non-ATPase regulatory subunit 1 OS=Homo sapiens OX=9606 GN=PSMD1 PE=1 SV=2 - [PSMD1_HUMAN] | 105.8 | 3 | 9.921E6 | 9.291E6 |  | 3 | - | 3.1 |
| Q9NUU7 | ATP-dependent RNA helicase DDX19A OS=Homo sapiens OX=9606 GN=DDX19A PE=1 SV=1 - [DD19A_HUMAN] | 53.9 | 3 | 9.308E6 | 6.022E6 |  | 3 | - | 3.1 |
| Q15007 | Pre-mRNA-splicing regulator WTAP OS=Homo sapiens OX=9606 GN=WTAP PE=1 SV=2 - [FL2D_HUMAN] | 44.2 | 3 | 9.091E6 | 7.715E6 |  | 3 | - | 3.1 |
| Q9H3P2 | Negative elongation factor A OS=Homo sapiens OX=9606 GN=NELFA PE=1 SV=3 - [NELFA_HUMAN] | 57.2 | 3 | 9.068E6 | 6.448E6 |  | 3 | - | 3.1 |
| Q13492 | Phosphatidylinositol-binding clathrin assembly protein OS=Homo sapiens OX=9606 GN=PICALM PE=1 SV=2 - [PICAL_HUMAN] | 70.7 | 3 | 9.040E6 | 1.426E7 |  | 3 | - | 3.1 |
| Q9NQZ2 | Something about silencing protein 10 OS=Homo sapiens OX=9606 GN=UTP3 PE=1 SV=1 - [SAS10_HUMAN] | 54.5 | 2 | 9.003E6 | 8.268E6 |  | 2 | - | 2.1 |
| Q86WB0 | Nuclear-interacting partner of ALK OS=Homo sapiens OX=9606 GN=ZC3HC1 PE=1 SV=1 - [NIPA_HUMAN] | 55.2 | 3 | 8.710E6 | 4.863E6 |  | 3 | - | 3.1 |
| P13489 | Ribonuclease inhibitor OS=Homo sapiens OX=9606 GN=RNH1 PE=1 SV=2 - [RINI_HUMAN] | 49.9 | 2 | 8.474E6 | 5.947E6 |  | 2 | - | 2.1 |
| Q9UKS7 | Zinc finger protein Helios OS=Homo sapiens OX=9606 GN=IKZF2 PE=1 SV=2 - [IKZF2_HUMAN] | 59.5 | 3 | 8.349E6 | 1.276E7 |  | 3 | - | 3.1 |
| P26640 | Valine--tRNA ligase OS=Homo sapiens OX=9606 GN=VARS PE=1 SV=4 - [SYVC_HUMAN] | 140.4 | 3 | 8.097E6 | 5.434E6 |  | 3 | - | 3.1 |
| P10768 | S-formylglutathione hydrolase OS=Homo sapiens OX=9606 GN=ESD PE=1 SV=2 - [ESTD_HUMAN] | 31.4 | 4 | 7.732E6 | 1.016E7 |  | 4 | - | 4.1 |
| Q9H967 | WD repeat-containing protein 76 OS=Homo sapiens OX=9606 GN=WDR76 PE=1 SV=2 - [WDR76_HUMAN] | 69.7 | 2 | 7.617E6 | 8.564E6 |  | 2 | - | 2.1 |
| O60684 | Importin subunit alpha-7 OS=Homo sapiens OX=9606 GN=KPNA6 PE=1 SV=1 - [IMA7_HUMAN] | 60.0 | 5 | 6.959E6 | 1.099E7 |  | 3 | - | 3.1 |
| Q9Y6A4 | Cilia- and flagella-associated protein 20 OS=Homo sapiens OX=9606 GN=CFAP20 PE=1 SV=1 - [CFA20_HUMAN] | 22.8 | 2 | 6.684E6 | 9.568E6 |  | 2 | - | 2.1 |
| Q8N2W9 | E3 SUMO-protein ligase PIAS4 OS=Homo sapiens OX=9606 GN=PIAS4 PE=1 SV=1 - [PIAS4_HUMAN] | 56.5 | 2 | 6.389E6 | 5.213E6 |  | 2 | - | 2.1 |
| Q7Z7H8 | 39S ribosomal protein L10, mitochondrial OS=Homo sapiens OX=9606 GN=MRPL10 PE=1 SV=3 - [RM10_HUMAN] | 29.3 | 3 | 6.312E6 | 6.239E6 |  | 3 | - | 3.1 |
| Q9UBX3 | Mitochondrial dicarboxylate carrier OS=Homo sapiens OX=9606 GN=SLC25A10 PE=1 SV=2 - [DIC_HUMAN] | 31.3 | 4 | 6.077E6 | 7.055E6 |  | 4 | - | 4.1 |
| P52306 | Rap1 GTPase-GDP dissociation stimulator 1 OS=Homo sapiens OX=9606 GN=RAP1GDS1 PE=1 SV=3 - [GDS1_HUMAN] | 66.3 | 3 | 5.485E6 | 4.788E6 |  | 3 | - | 3.1 |
| Q9Y6X3 | MAU2 chromatid cohesion factor homolog OS=Homo sapiens OX=9606 GN=MAU2 PE=1 SV=2 - [SCC4_HUMAN] | 69.0 | 4 | 5.283E6 | 5.783E6 |  | 4 | - | 4.1 |
| Q96I25 | Splicing factor 45 OS=Homo sapiens OX=9606 GN=RBM17 PE=1 SV=1 - [SPF45_HUMAN] | 44.9 | 5 | 5.258E6 | 6.485E6 |  | 5 | - | 5.2 |
| P40763 | Signal transducer and activator of transcription 3 OS=Homo sapiens OX=9606 GN=STAT3 PE=1 SV=2 - [STAT3_HUMAN] | 88.0 | 3 | 4.149E6 | 6.258E6 |  | 3 | - | 3.1 |
| Q96RL1 | BRCA1-A complex subunit RAP80 OS=Homo sapiens OX=9606 GN=UIMC1 PE=1 SV=2 - [UIMC1_HUMAN] | 79.7 | 3 | 3.353E6 | 5.377E6 |  | 3 | - | 3.1 |
| Q14966 | Zinc finger protein 638 OS=Homo sapiens OX=9606 GN=ZNF638 PE=1 SV=2 - [ZN638_HUMAN] | 220.5 | 2 | 2.080E6 | 3.545E6 |  | 2 | - | 2.1 |
| P28074 | Proteasome subunit beta type-5 OS=Homo sapiens OX=9606 GN=PSMB5 PE=1 SV=3 - [PSB5_HUMAN] | 28.5 | 3 | 1.403E6 | 3.088E6 |  | 3 | - | 3.1 |
| P27816 | Microtubule-associated protein 4 OS=Homo sapiens OX=9606 GN=MAP4 PE=1 SV=3 - [MAP4_HUMAN] | 120.9 | 3 | 1.010E6 | 5.509E6 |  | 3 | - | 3.1 |
| Q12830 | Nucleosome-remodeling factor subunit BPTF OS=Homo sapiens OX=9606 GN=BPTF PE=1 SV=3 - [BPTF_HUMAN] | 338.1 | 2 | 0.000E0 | 1.642E7 |  | 2 | - | 2.1 |
| Q7Z6Z7 | E3 ubiquitin-protein ligase HUWE1 OS=Homo sapiens OX=9606 GN=HUWE1 PE=1 SV=3 - [HUWE1_HUMAN] | 481.6 | 2 | 0.000E0 | 2.811E6 |  | 2 | - | 2.1 |
| Q9H0H5 | Rac GTPase-activating protein 1 OS=Homo sapiens OX=9606 GN=RACGAP1 PE=1 SV=1 - [RGAP1_HUMAN] | 71.0 | 6 | 0.000E0 | 5.796E6 |  | 6 | - | 6.2 |
| Q86U42 | Polyadenylate-binding protein 2 OS=Homo sapiens OX=9606 GN=PABPN1 PE=1 SV=3 - [PABP2_HUMAN] | 32.7 | 3 | 0.000E0 | 2.348E7 |  | 3 | - | 3.1 |
| Q15542 | Transcription initiation factor TFIID subunit 5 OS=Homo sapiens OX=9606 GN=TAF5 PE=1 SV=3 - [TAF5_HUMAN] | 86.8 | 3 | 0.000E0 | 6.513E6 |  | 3 | - | 3.1 |
| O95373 | Importin-7 OS=Homo sapiens OX=9606 GN=IPO7 PE=1 SV=1 - [IPO7_HUMAN] | 119.4 | 2 | 0.000E0 | 2.695E6 |  | 2 | - | 2.1 |
| P27824 | Calnexin OS=Homo sapiens OX=9606 GN=CANX PE=1 SV=2 - [CALX_HUMAN] | 67.5 | 2 | 0.000E0 | 1.489E7 |  | 2 | - | 2.1 |
| P46379 | Large proline-rich protein BAG6 OS=Homo sapiens OX=9606 GN=BAG6 PE=1 SV=2 - [BAG6_HUMAN] | 119.3 | 3 | 0.000E0 | 5.122E6 |  | 3 | - | 3.1 |
| O95202 | Mitochondrial proton/calcium exchanger protein OS=Homo sapiens OX=9606 GN=LETM1 PE=1 SV=1 - [LETM1_HUMAN] | 83.3 | 2 | 0.000E0 | 1.067E7 |  | 2 | - | 2.1 |
| Q9NY12 | H/ACA ribonucleoprotein complex subunit 1 OS=Homo sapiens OX=9606 GN=GAR1 PE=1 SV=1 - [GAR1_HUMAN] | 22.3 | 3 | 0.000E0 | 1.119E7 |  | 3 | - | 3.1 |
| P46063 | ATP-dependent DNA helicase Q1 OS=Homo sapiens OX=9606 GN=RECQL PE=1 SV=3 - [RECQ1_HUMAN] | 73.4 | 2 | 0.000E0 | 1.246E7 |  | 2 | - | 2.1 |
| P08708 | 40S ribosomal protein S17 OS=Homo sapiens OX=9606 GN=RPS17 PE=1 SV=2 - [RS17_HUMAN] | 15.5 | 3 | 0.000E0 | 5.157E6 |  | 3 | - | 3.1 |
| Q15102 | Platelet-activating factor acetylhydrolase IB subunit gamma OS=Homo sapiens OX=9606 GN=PAFAH1B3 PE=1 SV=1 - [PA1B3_HUMAN] | 25.7 | 3 | 0.000E0 | 1.347E7 |  | 3 | - | 3.1 |
| Q14004 | Cyclin-dependent kinase 13 OS=Homo sapiens OX=9606 GN=CDK13 PE=1 SV=2 - [CDK13_HUMAN] | 164.8 | 2 | 0.000E0 | 6.476E5 |  | 2 | - | 2.1 |
| Q9H0H0 | Integrator complex subunit 2 OS=Homo sapiens OX=9606 GN=INTS2 PE=1 SV=2 - [INT2_HUMAN] | 134.2 | 3 | 0.000E0 | 2.877E6 |  | 3 | - | 3.1 |
| Q6NUQ4 | Transmembrane protein 214 OS=Homo sapiens OX=9606 GN=TMEM214 PE=1 SV=2 - [TM214_HUMAN] | 77.1 | 2 | 0.000E0 | 1.001E7 |  | 2 | - | 2.1 |
| Q8TF68 | Zinc finger protein 384 OS=Homo sapiens OX=9606 GN=ZNF384 PE=1 SV=2 - [ZN384_HUMAN] | 63.2 | 4 | 0.000E0 | 5.952E6 |  | 4 | - | 4.1 |
| O75489 | NADH dehydrogenase [ubiquinone] iron-sulfur protein 3, mitochondrial OS=Homo sapiens OX=9606 GN=NDUFS3 PE=1 SV=1 - [NDUS3_HUMAN] | 30.2 | 3 | 0.000E0 | 8.471E6 |  | 3 | - | 3.1 |
| Q9NUW8 | Tyrosyl-DNA phosphodiesterase 1 OS=Homo sapiens OX=9606 GN=TDP1 PE=1 SV=2 - [TYDP1_HUMAN] | 68.4 | 2 | 0.000E0 | 1.309E7 |  | 2 | - | 2.1 |
| P50213 | Isocitrate dehydrogenase [NAD] subunit alpha, mitochondrial OS=Homo sapiens OX=9606 GN=IDH3A PE=1 SV=1 - [IDH3A_HUMAN] | 39.6 | 3 | 0.000E0 | 8.539E6 |  | 3 | - | 3.1 |
| P24666 | Low molecular weight phosphotyrosine protein phosphatase OS=Homo sapiens OX=9606 GN=ACP1 PE=1 SV=3 - [PPAC_HUMAN] | 18.0 | 3 | 0.000E0 | 9.786E6 |  | 3 | - | 3.1 |
| P49589 | Cysteine--tRNA ligase, cytoplasmic OS=Homo sapiens OX=9606 GN=CARS PE=1 SV=3 - [SYCC_HUMAN] | 85.4 | 2 | 0.000E0 | 4.233E6 |  | 2 | - | 2.1 |
| Q9Y483 | Metal-response element-binding transcription factor 2 OS=Homo sapiens OX=9606 GN=MTF2 PE=1 SV=3 - [MTF2_HUMAN] | 67.0 | 3 | 0.000E0 | 4.705E6 |  | 3 | - | 3.1 |
| Q06330 | Recombining binding protein suppressor of hairless OS=Homo sapiens OX=9606 GN=RBPJ PE=1 SV=3 - [SUH_HUMAN] | 55.6 | 2 | 0.000E0 | 7.752E6 |  | 2 | - | 2.1 |
| Q15554 | Telomeric repeat-binding factor 2 OS=Homo sapiens OX=9606 GN=TERF2 PE=1 SV=3 - [TERF2_HUMAN] | 59.6 | 7 | 0.000E0 | 1.113E7 |  | 7 | - | 7.2 |
| Q9NXE4 | Sphingomyelin phosphodiesterase 4 OS=Homo sapiens OX=9606 GN=SMPD4 PE=1 SV=3 - [NSMA3_HUMAN] | 97.7 | 4 | 0.000E0 | 9.765E6 |  | 4 | - | 4.1 |
