# Supplementary material for: Increased Expression of Long Noncoding RNA LOC100506314 in T cells from Patients with Nonsegmental Vitiligo and Its Contribution to Vitiligo Pathogenesis
Source: Mediators Inflamm. 2023 Sep 12;2023:2440377. doi: 10.1155/2023/2440377 (PMC10509001; doi:10.1155/2023/2440377)

Figure S1. Melt curve analysis for *Lnc-ARRDC3-1, PLCG1, A_33_P3229958,* *TM4SF19,* *WBP2NL, IFI27, IL17RB, CD1A, FPR2, CD1B, OAS3, OLFM1, SELP, MIR221, TERM1, RAB13, LOC100506314,* and *LOC101060810* transcripts. Representative image for melt curve analysis for (A) *Lnc-ARRDC3-1* transcripts, (B) *PLCG1* transcripts, (C) *A_33_P3229958* transcripts, (D) *TM4SF19* transcripts, (E) *WBP2NL* transcripts, (F) *IFI27* transcripts, (G) *IL17RB* transcripts, (H) *CD1A* transcripts, (I) *FPR2* transcripts, (J) *CD1B* transcripts, (K) *OAS3* transcripts*,* (L) *OLFM1* transcripts, (M) *SELP* transcripts, (N) *MIR221* transcripts, (O) *TERM1* transcripts and (P) *RAB13* transcripts, (Q) *LOC100506314* transcripts, and (R) *LOC101060810* transcripts.


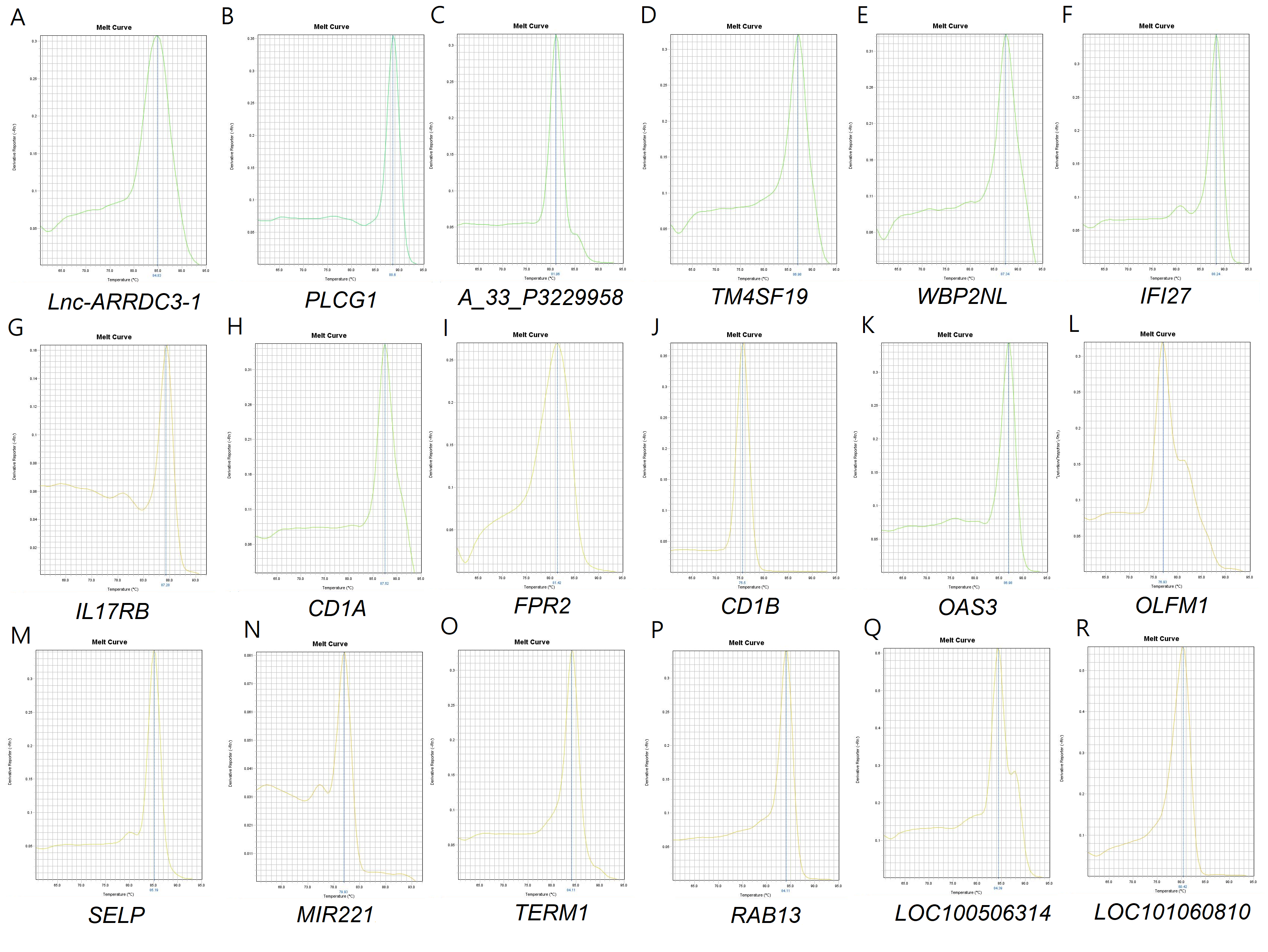

Supplement: Supplementary 2 — Melt curve analysis for Lnc-ARRDC3-1, PLCG1, A_33_P3229958, TM4SF19, WBP2NL, IFI27, IL17RB, CD1A, FPR2, CD1B, OAS3, OLFM1, SELP, MIR221, TERM1, RAB13, LOC100506314, and LOC101060810 transcripts. Representative image for melt curve analysis for (A) Lnc-ARRDC3-1 transcripts, (B) PLCG1 transcripts, (C) A_33_P3229958 transcripts, (D) TM4SF19 transcripts, (E) WBP2NL transcripts, (F) IFI27 transcripts, (G) IL17RB transcripts, (H) CD1A transcripts, (I) FPR2 transcripts, (J) CD1B transcripts, (K) OAS3 transcripts, (L) OLFM1 transcripts, (M) SELP transcripts, (N) MIR221 transcripts, (O) TERM1 transcripts and (P) RAB13 transcripts, (Q) LOC100506314 transcripts, and (R) LOC101060810 transcripts. [file 2440377.f2.doc]
